# Neurosurgery compared to orthopedic spine consultation: A single level I trauma center experience

**DOI:** 10.1016/j.bas.2024.102808

**Published:** 2024-04-05

**Authors:** Shaina Sedighim, Brynn Sargent, Areg Grigorian, Christina Grabar, Anvesh R. Macherla, Michael Oh, Yu-Po Lee, John Scolaro, Jefferson Chen, Jeffry Nahmias

**Affiliations:** a Division of Trauma, Burns, And Surgical Critical Care, Department of Surgery, University of California, Irvine, Orange, CA, USA; b Department of Neurosurgery, University of California, Irvine, Orange, CA, USA; c Department of Orthopedic Surgery, University of California, Irvine, Orange, CA, USA

**Keywords:** Spine surgery, Orthopedic surgery, Neurosurgery, Vertebral fractures, Traumatic spine injuries

## Abstract

**Introduction:**

Both Orthopedic Surgery (OS) and Neurosurgery (NS) perform spine surgery in the setting of trauma. However, it is unknown whether outcomes differ between these specialties. This study compares management and outcomes for vertebral fractures between NS and OS, hypothesizing similar operation rate, length of stay (LOS), and readmission.

**Research question:**

Do outcomes differ between NS and OS in the management of vertebral fractures following trauma?

**Methods:**

A retrospective single-center study was conducted on adult patients with cervical, thoracic, lumbar, and sacral fractures treated at a single trauma center, where no standardized pathway exists across NS and OS. Patients were compared for injury profile, diagnostic imaging, and operative techniques as well as LOS, mortality, and complications.

**Results:**

A total of 630 vertebral fracture patients (OS:350 (55.6%); NS:280 (44.4%)) were included. NS utilized magnetic resonance imaging (MRI) more commonly (36.4% vs. 22.6%, p < 0.001). NS patients more often underwent operation (13.2% vs. 7.4%, p = 0.016) despite similar fracture number and severity (p > 0.05). Post-operative complications, LOS, and readmission rates were similar between cohorts (p > 0.05).

**Discussion and conclusion:**

Despite similar injury profiles, NS had higher rates of MRI usage and operative interventions in the context of traumatic spine fractures. Despite differences in management, major clinical outcomes were similar between NS and OS. However, we do call for further standardization of evaluation and treatment of patients based on established algorithms from such as the AOSpine Thoracolumbar Spine Injury Classification System (ATLICS).

## Highlights

• There were similar outcomes in this cohort for vertebral fractures managed by neurosurgery and orthopedics.
• Neurosurgery more frequently used MRI and chose operative intervention as compared to orthopedics.
• Future research is needed to further standardize spine fracture management.

## 1 Introduction

Spine injuries, affecting 1–6% of trauma patients in the United States (Greenbaum et al., 2009; Bizimungu et al., 2020), are primarily managed by Neurosurgery (NS) or Orthopedic Surgery (OS) specialists (Lad et al., 2021; Daniels et al., 2014). Despite both specialties requiring a minimum of five years of surgical training, discrepancies exist in their spinal surgery experience. Recent data indicate that NS residents are significantly more involved in spinal surgeries, accumulating substantially more surgical hours compared to OS residents (Lad et al., 2021).

While NS residents express higher confidence in performing spinal procedures, assessments of competency between NS and OS practitioners reveal similarities in managing spinal pathologies and post-operative complications (Dvorak et al., 2006). However, differences in clinical decision-making, surgical interventions, and outcomes emerge between the two specialties, leading to variations in procedures and diagnostic approaches (Pejrona et al., 2018).

At our Level-I trauma center, both NS and OS specialists rotate spine surgery calls, with observed comparable care standards and outcomes for traumatic spinal injuries. Despite limited clinical data on traumatic spinal injuries in the United States, this study aims to scrutinize differences in management, operative approaches, and outcomes for vertebral body fractures handled by OS versus NS specialists. This investigation hypothesizes comparable incidences of operations, length of stay (LOS), and readmission rates between the two specialties in managing traumatic vertebral body fractures.

## 2 Methods

**Fig. 1:**
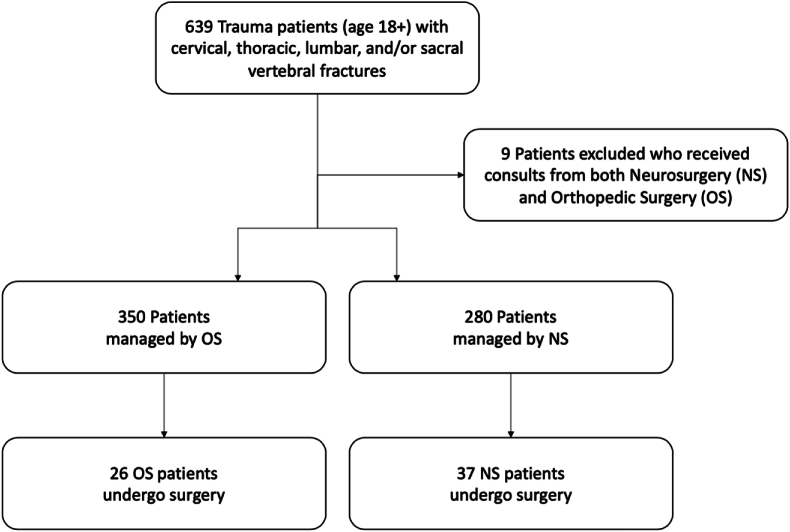
Flowchart of patient study inclusion Fig. 1: Arrows describe how patients were categorized based on the consulting service and management. NS = Neurosurgery. OS = Orthopedic Surgery.

This study was performed in line with the principles of the Declaration of Helsinki. Approval was granted by the Institutional Review Board of the University of California, Irvine (IRB #20195334). Subsequently, a retrospective analysis between October 2017 and September 2019 of adult (18 years-old or older) trauma patients with cervical, thoracic, lumbar, and/or sacral vertebral fractures at a single urban academic level-I trauma center was performed. Number of fractures and specific fracture type were determined by an attending radiologist using available diagnostic imaging studies, including computed tomography (CT) imaging which is standard imaging for all adult patients with spine fractures at our institution. During the time of our study, there was no established protocol or guideline, such as the Thoracolumbar Injury Classification and Severity Score (TLICS) (Vaccaro et al., 2005) or the AO Spine Classification System, (AOSpine) (Kepler et al., 2016) governing the treatment approach across both services. As such, treatment decisions were made by the attending surgeon based on their clinical judgment and the individual patient's presentation. Patients who received NS consultation were compared to patients who received OS spine consultation. Patients who received spine-related consultations from both services were excluded (Fig. 1). All these patients had complex sacral fractures ± pelvic fractures and were managed by OS.

The primary outcomes of interest were LOS, 30-day readmission rates, and whether the patient underwent operative intervention. Demographic data collected included age, sex (self-reported), and body mass index (BMI). Comorbidities included congestive heart failure, coronary artery disease, cerebrovascular accident, myocardial infarction, hypertension, diabetes, chronic obstructive pulmonary disease, cirrhosis, end-stage renal disease, and psychiatric illness as well as previous history of spinal injury or surgery. Injury profile was characterized by mechanism of injury, number of fractures, fracture location, type of fracture (i.e., compression, burst, burst/compression, unilateral and bilateral facet, perched, and jumped), injury severity score (ISS), abbreviated injury scale (AIS) for the spine, and presence of neurological deficit.

Clinical variables measured included the specific type of diagnostic imaging (computed tomography (CT), magnetic resonance imaging (MRI), or X-ray) and use of supportive brace. Surgical details collected included vertebral level of injury, operative approach (e.g., anterior or posterior), graft material (e.g., autograft and/or allograft), time to surgery, and total operative time in hours. Additional outcomes collected included intensive care unit (ICU) LOS, in-hospital mortality, discharge disposition (i.e. home, skilled nursing facility (SNF), acute rehabilitation unit, and long-term acute care facility), return to the hospital Emergency Department but not readmitted, return to the operating room, and post-operative complications. Measured complications included hemorrhage, surgical site infection, deep venous thrombosis, pulmonary embolism, sepsis, pneumonia, and acute respiratory distress syndrome. Outcomes were evaluated after hospitalization and 30 days post-discharge via electronic medical record review.

Descriptive statistics were performed for all variables. Continuous variables were compared using a Mann-Whitney-U test and categorical variables were compared using a chi-square test. Categorical data was reported as percentages and continuous data was reported as medians with interquartile range. All p-values were two-sided with α < 0.05. Analysis was performed using IBM SPSS Statistics for Windows (Version 24, IBM Corp., Armonk, NY).

## 3 Results

### 3.1 Demographics and comorbidities

A total of 630 patients with vertebral fractures were included with 350 (55.6%) managed by OS and 280 (44.4%) managed by NS. Patient demographics including age, sex, and BMI were similar between the two cohorts (all p > 0.05). History of major medical comorbidities and prior spinal injury or surgery were also similar between cohorts (all p > 0.05) except for increased cirrhosis (2.0% vs. 0.0%, p = 0.017) in OS patients (Table 1).

**Table 1:** Demographics of patients presenting with vertebral fractures stratified by consulting service.

Characteristic	Orthopedic Surgery (n = 350)	Neurosurgery (n = 280)	p-value
Age, year, median (IQR)	54 (35, 70)	54 (34, 65)	0.390
Male, n (%)	213 (60.9%)	183 (65.4%)	0.245
BMI, median (IQR)	25.8 (22.6, 29.2)	25.1 (22.3, 28.9)	0.288
Comorbidities, n (%)			
Congestive heart failure	9 (2.6%)	13 (4.6%)	0.159
Coronary artery disease	8 (2.3%)	8 (2.9%)	0.651
Cerebrovascular accident	11 (3.1%)	5 (1.8%)	0.282
Myocardial infarction	4 (1.1%)	4 (1.4%)	0.750
Hypertension	98 (28.0%)	75 (26.8%)	0.734
Diabetes	40 (11.4%)	40 (14.3%)	0.285
COPD	11 (3.1%)	6 (2.1%)	0.441
Cirrhosis	7 (2.0%)	0 (0.0%)	**0.017**
End-stage renal disease	10 (2.9%)	6 (2.1%)	0.571
Psychiatric illness	29 (8.3%)	32 (11.4%)	0.185
Prior spine injury, n (%)	22 (6.3%)	15 (5.4%)	0.622
Prior spine surgery, n (%)	14 (4.0%)	14 (5.0%)	0.545

BMI = body mass index, COPD = chronic obstructive pulmonary disease, IQR = interquartile range.

### 3.2 Injury profile

The two cohorts were similar in terms of mechanism of injury, number of fractures, ISS, spine AIS, and presence of focal neurological deficits at presentation. Regarding fracture location, compared to NS, OS patients more commonly sustained sacral fractures (11.1% vs. 4.3%, p = 0.002) whereas cervical, thoracic, and lumbar fractures were similar between cohorts. In terms of fracture patterns there was no difference in types of fractures including burst, compression, burst/compression, unilateral and bilateral facet, perched, and jumped facet (all p > 0.05) (Table 2).

**Table 2:** Injury profile and clinical work-up of patients presenting with vertebral fractures stratified by consulting service.

Characteristic	Orthopedic Surgery (n = 350)	Neurosurgery (n = 280)	p-value
Mechanism of injury, n (%)			
Blunt	343 (98.0%)	271 (96.8%)	0.336
Fall	124 (35.4%)	85 (30.4%)	0.179
MVC	121 (34.6%)	112 (40.0%)	0.161
Auto vs pedestrian/bike	65 (18.6%)	47 (16.8%)	0.560
Motorcycle crash	26 (7.4%)	19 (6.8%)	0.756
Number of spinal fractures, median (IQR)	2 (1, 3)	2 (1, 3)	0.674
Level of spine involved, n (%)			
Cervical spine	126 (36.0%)	98 (35.0%)	0.794
Thoracic spine	168 (48.0%)	145 (51.8%)	0.345
Lumbar spine	148 (42.3%)	118 (42.1%)	0.971
Sacral spine	39 (11.1%)	12 (4.3%)	**0.002**
Type of fracture, n (%)			
Compression	132 (37.7%)	105 (37.5%)	0.956
Burst	20 (5.7%)	13 (4.6%)	0.549
Burst/Compression	1 (0.3%)	5 (1.8%)	0.054
Unilateral facet	18 (5.1%)	18 (6.4%)	0.490
Bilateral facet	3 (0.9%)	4 (1.4%)	0.497
Perched	5 (1.4%)	2 (0.7%)	0.395
Jumped	0 (0.0%)	3 (1.1%)	0.052
ISS, median (IQR)	14 (8, 22)	12 (9, 22)	0.570
AIS spine, median (IQR)	2 (2, 2)	2 (2, 2)	0.552
Neurological deficit, n (%)	31 (8.9%)	29 (10.4%)	0.524
Upper extremity motor	18 (5.1%)	13 (4.6%)	0.773
Upper extremity sensory	12 (3.4%)	4 (1.4%)	0.113
Lower extremity motor	21 (6.0%)	26 (9.3%)	0.119
Lower extremity sensory	15 (4.3%)	16 (5.7%)	0.410
Imaging studies, n (%)			
CT	350 (100.0%)	280 (100.0%)	
MRI	79 (22.6%)	102 (36.4%)	**<0.001**
X-Ray	270 (77.1%)	223 (79.6%)	0.450
Spinal surgery, n (%)	26 (7.4%)	37 (13.2%)	**0.016**
Brace, n (%)	201 (57.4%)	149 (53.2%)	0.290

MVC = motor vehicle collision, IQR = interquartile range, ISS = injury severity score, AIS = abbreviated injury scale, CT = computed tomography, MRI = magnetic resonance imaging.

### 3.3 Clinical management

In terms of imaging studies, NS utilized MRI more often than OS (36.4% vs. 22.6%, p < 0.001) whereas CT and X-ray were used at a similar rate across specialties (all p > 0.05). Spine bracing was recommended for a similar majority amongst cohorts (53.2% vs. 57.4%, p = 0.290). Patients managed by NS more commonly underwent operation prior to discharge (13.2% vs. 7.4%, p = 0.016) (Table 2).

OS more commonly utilized an anterior surgical approach (26.9% vs. 8.1%, p = 0.044) whereas NS more commonly used a posterior approach (89.2% vs. 69.2%, p = 0.047). Time to surgery, operative time, and use of intraoperative imaging and monitoring were otherwise similar between cohorts (all p > 0.05). However, NS more frequently operated on thoracic spine fractures (75.7% vs. 42.3%, p = 0.007) whereas OS more frequently operated on sacral spine fractures (23.1% vs. 2.7%, p = 0.013). In patients managed surgically, autografts were more commonly used by NS (59.5% vs. 11.5%, p < 0.001), while allografts were used at a similar rate between cohorts (67.6% vs. 61.5%, p = 0.621) (Table 3).

**Table 3:** Operative management of patients presenting with vertebral fractures stratified by consulting service.

Characteristic	Orthopedic Surgery (n = 26)	Neurosurgery (n = 37)	p-value
Surgery vertebral level, n (%)			
Cervical spine	8 (30.8%)	12 (32.4%)	0.889
Thoracic spine	11 (42.3%)	28 (75.7%)	**0.007**
Lumbar spine	12 (46.2%)	12 (32.4%)	0.270
Sacral spine	6 (23.1%)	1 (2.7%)	**0.011**
Surgical approach, n (%)			
Anterior	7 (26.9%)	3 (8.1%)	**0.044**
Posterior	18 (69.2%)	33 (89.2%)	**0.047**
Intraoperative monitoring, n (%)	23 (88.5%)	33 (89.2%)	0.928
Intraoperative imaging, n (%)	24 (92.3%)	36 (97.3%)	0.360
Allograft, n (%)	16 (61.5%)	25 (67.6%)	0.621
Autograft, n (%)	3 (11.5%)	22 (59.5%)	**<0.001**
Time to surgery, hrs, median (IQR)	45 (27, 88)	62 (22, 97)	0.933
Operative time, hrs, median (IQR)	4 (3, 5)	4 (3, 6)	0.475
Neurological deficit, n (%)	7 (26.9%)	16 (43.2%)	0.185
Complications, n (%)			
Hemorrhage	0 (0.0%)	0 (0.0%)	
Surgical site infection (deep or superficial)	0 (0.0%)	0 (0.0%)	
Deep venous thrombosis	0 (0.0%)	2 (5.4%)	0.228
Pulmonary embolism	0 (0.0%)	0 (0.0%)	
ARDS	0 (0.0%)	4 (10.8%)	0.083
Sepsis	0 (0.0%)	2 (5.4%)	0.228
Pneumonia	0 (0.0%)	5 (13.5%)	0.051

IQR = interquartile range, ARDS = acute respiratory distress syndrome.

### 3.4 Outcomes

NS and OS patients had similar LOS, readmissions, in-hospital mortality, and rate of return to the operating room (all p > 0.05). All post-operative complications were similar between cohorts (all p > 0.05). Discharge disposition was also similar between cohorts (Table 4).

**Table 4:** Clinical outcomes and discharge disposition of patients presenting with vertebral fractures stratified by consulting service.

Characteristic	Orthopedic Surgery (n = 350)	Neurosurgery (n = 280)	p-value
In-hospital mortality, n (%)	17 (4.9%)	10 (3.6%)	0.429
Hospital LOS, median (IQR)	5 (2, 10)	5 (3, 10)	0.508
ICU LOS, median (IQR)	0 (0, 3)	0 (0, 4)	0.503
Discharge disposition, n (%)			
Home	182 (52.0%)	149 (53.2%)	0.762
Skilled nursing facility	58 (16.6%)	45 (16.1%)	0.866
Acute rehabilitation unit	38 (10.9%)	34 (12.1%)	0.614
Long term acute care facility	32 (9.1%)	28 (10.0%)	0.716
30-day return to ED, n (%)	32 (9.1%)	36 (12.9%)	0.135
30-day readmission, n (%)	16 (4.6%)	17 (6.1%)	0.401
30-day return to OR, n (%)	4 (1.1%)	0 (0.0%)	0.073

IQR = interquartile range, LOS = length of stay, ICU = intensive care unit, ED = emergency department, OR = operating room.

## 4 Discussion

In the United States, traumatic vertebral fractures are managed by NS and OS physicians (Lad et al., 2021; Daniels et al., 2014). This two-year analysis of vertebral fractures at a single level I trauma center found similar patient demographics, injury profiles, and neurological deficits between patients managed by NS and OS. However, NS patients underwent MRI, surgery, and autograft more frequently compared to OS. Despite these differences there was no difference in other patient outcomes including mortality, LOS, and readmissions. To the best of our knowledge, this is the first study which directly compares clinical management and outcomes of vertebral fractures managed by NS and OS in the acute trauma setting.

In many medical scenarios, multiple specialties may care for a shared patient population. When this occurs, it is important to ensure that quality of care persists across these specialties. Moreover, handling trauma cases can be demanding in terms of the intensive time and effort required for acute injury management (Heponiemi et al., 2015). Previous studies have advocated for a similar system of alternating specialty call for other traumatic injuries such as facial fractures (Christian et al., 2022; Susarla et al., 2016). Hence, it is perhaps not surprising that our study observed comparable outcomes for patients under the care of NS and OS. Notably, the NS and OS spine teams at our hospital work closely together, with residents cross-rotating services as well as participating in operative cases together. Continued cross-specialty collaboration, both in the clinical as well as the educational aspects of residency training will hopefully continue to promote equivalent care and outcomes for patients with traumatic spinal injuries. Some experts have proposed an increase integration of cross-specialty collaboration during residency training as a means to narrow the gap in case numbers and operative time between OS and NS residency programs (Lad et al., 2021).

The decision to recommend surgical intervention for traumatic spine injury is certainly multifactorial and requires significant counseling and shared decision making to be conducted optimally (Skou et al., 2020; Tan et al., 2022). Despite similar demographics and injury profiles, this current study demonstrated increased rate of index hospitalization surgical intervention by NS compared to OS. One potential explanation for this difference may be increased identification of unstable ligamentous injury recognized by NS with their increased use of MRI compared to OS. The majority of this difference was observed in cases involving thoracic spine injuries. NS may have greater comfort or familiarity with thoracic corpectomy or transpedicular decompression, however the underlying reason for this difference is not completely clear at this time. Regardless, it should be noted that this intervention did not appear to impact LOS, mortality, or readmissions and thus appears to be safe for patients. Future prospective studies including long-term data and patient reported outcomes in this sub-population of thoracic spine injuries may help elucidate if the higher rate of operation leads to improved quality of life or places patients at unnecessary risk.

This study may help promote the need for standardized training programs in the field of spinal surgery. Initiatives such as AOSpine (Global Spine Diploma Exam) hold the potential to bridge training disparities across specialties, harmonizing knowledge and honing skills to ensure uniformity in treatment approaches. Such standardization plays a pivotal role in enhancing patient outcomes by reducing treatment variability and ensuring optimal care is provided.

Our study has many limitations including those inherent to its retrospective design such as reliance on retrospective electronic medical record documentation that may have missing information and/or misclassification. Additionally, as a single center study it lacks generalizability. The comparison of operative techniques and post-operative complications is also constrained by the relatively small sample size of patients who underwent surgical intervention. Also, comparing NS and OS as distinct entities may be restricted by their close collaboration at our institution.

The absence of a standardized classification system for fracture subtypes may introduce variability in the interpretation of injuries, potentially limiting the generalizability of our findings across different medical settings. The significant difference in postoperative complications between orthopedic and neurosurgical approaches highlights the importance of establishing consistent guidelines to guide surgical decision-making and postoperative care. These findings emphasize the opportunity for improving patient outcomes through the development and implementation of evidence-based protocols aimed at standardizing assessment and treatment practices for spinal fractures. Furthermore, this study lacks key variables such as neurological function at discharge and patient-centered metrics including post-discharge quality of life and functional status. Additionally, we recognize that the absence of long-term follow-up data beyond 30 days is a limitation and restricts a comprehensive assessment of postoperative outcomes and the adequacy of preoperative decisions. Furthermore, the absence of patient-reported outcomes is a significant limitation and merits future prospective research. This study also does not include measurements of bone quality such as hounsefield units on CT imaging, which may impact perioperative decisions for older adults (e.g., 65 years of age and older). Finally, while outside of the scope of this retrospective study, a prospective multicenter evaluation correlating treatment strategies of NS and OS with established decision algorithms such as TLICS (Vaccaro et al., 2005) and AOSpine (Kepler et al., 2016) would be highly beneficial.

In conclusion, this single center study comparing alternating weeks of NS and OS consultations for spine injuries, revealed generally comparable outcomes in terms of LOS, mortality, and readmission rates. Nevertheless, NS-treated patients underwent MRI, surgery, and autograft procedures more frequently. Despite this, considering the absence of significant differences in major outcomes between the two groups, we believe this underscores the feasibility and safety of an alternating call schedule for spine injuries. Further research is warranted to assess the variation in surgical interventions and complications, particularly for thoracic and sacral spine injuries seen in this study. Adoption of standardized classification systems may help optimize care for these sub-populations within the realm of traumatic spine injuries. Furthermore, the variation in postoperative neurological deficits between the two cohorts (although not statistically significant) is noteworthy and further investigation is necessary to elucidate the reason for this trend.

## Funding

The authors did not receive support from any organization for the submitted work.

## Declaration of competing interest

The authors have no relevant financial or non-financial interests to disclose.
